# Effects of the Dietary Protein and Carbohydrate Ratio on Gut Microbiomes in Dogs of Different Body Conditions

**DOI:** 10.1128/mBio.01703-16

**Published:** 2017-01-24

**Authors:** Qinghong Li, Christian L. Lauber, Gail Czarnecki-Maulden, Yuanlong Pan, Steven S. Hannah

**Affiliations:** a Nestlé Purina Research, St. Louis, Missouri, USA; b Nestlé Institute of Health Science, Lausanne, Switzerland; Icahn School of Medicine at Mount Sinai

## Abstract

Obesity has become a health epidemic in both humans and pets. A dysbiotic gut microbiota has been associated with obesity and other metabolic disorders. High-protein, low-carbohydrate (HPLC) diets have been recommended for body weight loss, but little is known about their effects on the canine gut microbiome. Sixty-three obese and lean Labrador retrievers and Beagles (mean age, 5.72 years) were fed a common baseline diet for 4 weeks in phase 1, followed by 4 weeks of a treatment diet, specifically, the HPLC diet (49.4% protein, 10.9% carbohydrate) or a low-protein, high-carbohydrate (LPHC) diet (25.5% protein, 38.8% carbohydrate) in phase 2. 16S rRNA gene profiling revealed that dietary protein and carbohydrate ratios have significant impacts on gut microbial compositions. This effect appeared to be more evident in obese dogs than in lean dogs but was independent of breed. Consumption of either diet increased the bacterial evenness, but not the richness, of the gut compared to that after consumption of the baseline diet. Macronutrient composition affected taxon abundances, mainly within the predominant phyla, *Firmicutes* and *Bacteroidetes*. The LPHC diet appeared to favor the growth of *Bacteroides uniformis* and *Clostridium butyricum*, while the HPLC diet increased the abundances of *Clostridium hiranonis*, *Clostridium perfringens*, and *Ruminococcus gnavus* and enriched microbial gene networks associated with weight maintenance. In addition, we observed a decrease in the *Bacteroidetes* to *Firmicutes* ratio and an increase in the *Bacteroides* to *Prevotella* ratio in the HPLC diet-fed dogs compared to these ratios in dogs fed other diets. Finally, analysis of the effect of diet on the predicted microbial gene network was performed using phylogenetic investigation of communities by reconstruction of unobserved states (PICRUSt).

## IMPORTANCE

More than 50% of dogs are either overweight or obese in the United States. A dysbiotic gut microbiota is associated with obesity and other metabolic problems in humans. HPLC diets have been promoted as an effective weight loss strategy for many years, and potential effects were reported for both humans and dogs. In this study, we explored the influence of the protein and carbohydrate ratio on the gut microbiome in dogs with different body conditions. We demonstrated significant dietary effects on the gut microbiome, with greater changes in obese dogs than in lean dogs. The HPLC diet-fed dogs showed greater abundances of *Firmicutes* but fewer numbers of *Bacteroidetes* than other dogs. This knowledge will enable us to use prebiotics, probiotics, and other nutritional interventions to modulate the gut microbiota and to provide an alternative therapy for canine obesity.

## INTRODUCTION

The complex community of trillions of microbes densely populates the gastrointestinal tracts of their host and plays a significant role in an animal’s health and disease (1). The gut microbiota is involved in nutrient absorption, energy harvest, and metabolic homeostasis, among other things (2). Dysbiotic gut microbiotas have been associated with obesity, metabolic syndrome, cardiovascular disease, immune disorders, and liver and brain diseases (3–6). In both humans and mice, changes in the abundances of predominant gut microbes have been linked to excess body fat, with more *Firmicutes* and fewer *Bacteroidetes* in obese individuals than in lean ones (7–9). This change was reversible, as the *Bacteroidetes* to *Firmicutes* ratios (B/F ratios) in obese mice became similar to those in lean mice after diet-induced weight loss (7, 10). Shifts of gut microbial composition that favor increased *Firmicutes* abundance was thought to cause an increased efficiency in energy extraction from the diet, resulting in higher levels of short-chain fatty acids (SCFAs) that are suspected of altering the metabolism of obese individuals (8, 11, 12). Both genetic and environmental factors have significant impacts on the structure and composition of the gut microbiota, with diet being one of the greatest influences that can rapidly alter the gut microbiome (13–15). For instance, fecal microbiota of children from a rural African village, where the typical diet is rich in fiber, had increased *Bacteroidetes* but decreased *Firmicutes* abundances compared to Western children (16). Studies of humans suggested that gut microbial communities may be clustered into three enterotypes, as dominated and distinguished by three bacterial genera, *Bacteroides*, *Prevotella*, and *Ruminococcus* (17). The *Prevotella* enterotype was strongly associated with a greater intake of carbohydrates derived from plants, while the *Bacteroides* enterotype was associated with consumption of animal protein and fat (16, 18). However, new studies indicated that the boundaries between enterotypes may be fluid, not as well defined as previously reported (65).

In humans, obesity has become a public health epidemic in the past 3 decades (19). Similarly, obesity in pets has become more prevalent, and current studies suggest that 54% of dogs are overweight or obese (http://www.petobesityprevention.org). Hence, there is a significant interest in body weight management by modifying macronutrient distribution in diets. The concept of using high-protein, low-carbohydrate (HPLC) diets for body weight loss has been promoted as an effective weight loss strategy for many years (20). Potential benefits of HPLC diets, including increased satiety, reduced hunger, and preservation of lean body mass during body weight reduction, were reported for both humans and dogs (21–23). In rats, an HPLC diet appears to be effective in reducing both caloric intake and body weight gain (24). Our study also indicated that an HPLC diet significantly reduces weight gain in lean dogs without reducing the caloric intake (unpublished data), though the influences of the dietary protein-to-carbohydrate ratio on the gut microbiome in dogs remain unclear.

The microbiota of the canine gastrointestinal tract has only recently begun to be explored, as evidenced by the relatively small number of studies compared to the number of studies evaluating the human gut microbiome. Information on the effect of an HPLC diet on the gut microbiotas of pets is also scarce (25). The aim of this study was to monitor the impact of dietary macronutrient composition on the gut microbiome, using 16S rRNA gene sequencing, in lean and obese dogs of two breeds. Our hypothesis was that dietary protein-to-carbohydrate ratios alter gut microbiota compositions mainly within the predominant phyla, *Firmicutes* and *Bacteroides*. We expected to observe predicted gene networks for weight maintenance and metabolism and differential dietary impacts in obese versus lean dogs. This knowledge will enable us to modulate gut microbiomes using prebiotics, probiotics, or other nutritional-intervention approaches and to provide an alternative therapy for canine obesity or other metabolic disorders in the near future.

## RESULTS

### Feeding study and fecal DNA-sequencing analysis.

In phase 1, the mean body fat percentages for lean or normal (LN) and overweight or obese (OW) dogs, respectively, were 22.42% ± 1.24% (mean ± standard error) and 39.08% ± 1.30% for Beagles (*P* = 2.59e−10) and 25.68% ± 1.59% and 39.94% ± 1.07% for Labrador retrievers (*P* = 8.91e−08). At the end of phase 2, the mean body fat percentages for LN and OW dogs, respectively, were 18.18% ± 1.45% and 39.98% ± 1.40% for Beagles (*P* = 6.85e−12) and 22.51% ± 1.94% and 41.16% ± 0.83% for Labrador retrievers (*P* = 3.72e−08). No significant differences were observed in the mean ages and body fat values between HPLC and low-protein, high-carbohydrate (LPHC) diet-fed dogs within the phase and body condition groups (*P* > 0.30). There was no significant change in body fat between phase 1 and phase 2 except in the HPLC diet-fed LN dogs (*P* = 0.015) (Table 1).

**TABLE 1:** Physical characteristics of dogs enrolled in the study

Phenotype	Diet	Breed		Sex		Mean age (yr ± SE)	Mean body fat (% ± SE)^b^		*P* value^c^
		Beagle	Labrador retriever	Male	Female		Phase 1	Phase 2	
Lean or normal (LN)	HPLC	8	8^a^	9^a^	7	5.68 ± 0.73	24.84 ± 1.08	20.37 ± 1.37	0.015
	LPHC	8	8	9	7	5.79 ± 0.59	23.61 ± 1.69	20.39 ± 1.97	0.226
Overweight or obese (OW)	HPLC	8	8	9	7	5.46 ± 0.67	39.48 ± 1.25	39.78 ± 1.29	0.9869
	LPHC	8	8	7	9	5.9 ± 0.70	39.54 ± 1.14	41.36 ± 0.96	0.234

a One male Labrador retriever of the HPLC diet-fed LN group received an antibiotic in phase 2 and was excluded.

b Body fat was measured by dual-energy X-ray absorption (DEXA) after 4 weeks on the base diet (phase 1) and then again after 4 weeks on the experimental diets (phase 2). Body fat percentages within the phase and body condition groups did not differ (*P* > 0.30).

c Difference in body fat from phase 1 to phase 2. A *P* value of <0.05 indicates statistical significance.

One dog received antibiotic therapy toward the end of phase 2 due to skin infections prior to sample collection and was removed from the analysis. The phase 1 sample from a second dog was also excluded due to poor sequencing results. Hence, a total of three data points were excluded from analysis. A total of 6,011,483 high-quality 16S rRNA gene sequences were obtained for 125 samples, with an average of 48,092 ± 1,772 sequences per sample. The median sequence length was 422 bp, with a range between 357 bp and 449 bp.

### Dietary effects on gut microbial alpha and beta diversities in LN versus OW dogs.

**FIG 1:**
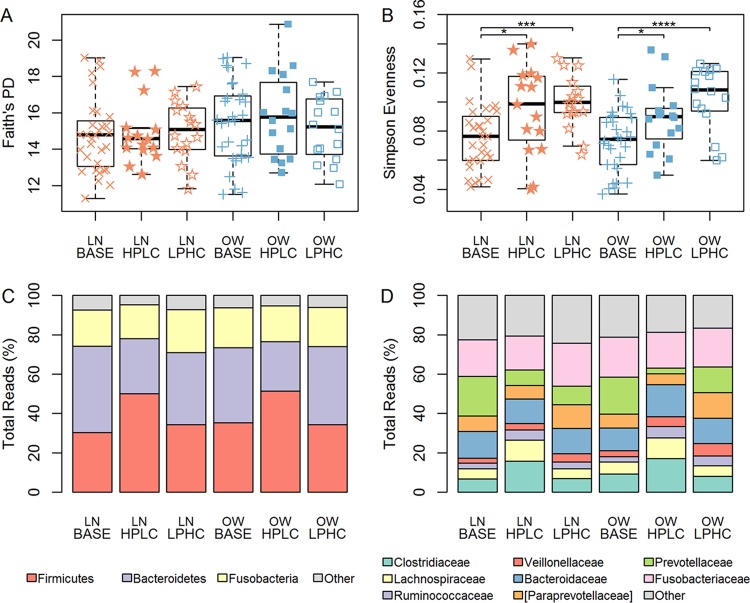
(A and B) Alpha diversity and (C and D) relative abundances of gut microbiota among base, HPLC, and LPHC dogs in LN vs. OW body condition groups. Alpha diversities were determined using (A) Faith's phylogenetic diversity (PD) and (B) Simpson's evenness by diet and body condition. Red indicates LN dogs, and blue indicates OW dogs. No difference in PD index was found. A significant difference was observed in Simpson’s evenness values between both HPLC and LPHC diet-fed dogs and base diet-fed dogs. However, the difference between HPLC and LPHC diet-fed dogs was not significant for either body condition group (*P* = 0.7405 and *P* = 0.0562, respectively). Levels of significance are denoted as follows: *, *P* < 0.05; **, *P* < 0.01; ***, *P* < 0.001; and ****, *P* < 0.0001. (C) Significant differences in phylum abundances were found between the protein-rich dietary group (HPLC diet) and the carbohydrate-rich dietary group (base or LPHC diet) for both *Firmicutes* and *Bacteroidetes* (*P* < 0.05) but not *Fusobacteria*. (D) With regard to family abundances, dogs fed a protein-rich diet had a significant increase in *Clostridiaceae* (teal) and *Lachnospiraceae* (yellow) compared with abundances in dogs fed a carbohydrate-rich diet, while *Prevotellaceae* (lime green) abundances were significantly different among all three groups (*P* < 0.001).

No significant difference in microbial richness using Faith’s phylogenetic diversity index was observed among base, HPLC, and LPHC diet-fed dogs in either the LN or the OW group (Fig. 1A). However, gut microbiota evenness (Simpson's evenness index) was significantly greater in HPLC and LPHC diet-fed dogs than in base diet-fed dogs (*P* < 0.05) (Fig. 1B). These diets were slightly higher in fiber than the base diet. The difference in Simpson's evenness indexes between HPLC and LPHC diet-fed dogs was not significant in either the OW group or the LN group (*P* = 0.0562 and *P* = 0.7405, respectively).

We used the weighted UniFrac metric to quantify differences in microbial community compositions and tested for significant effects of diet, breed, sex, body condition, and cohort using permutational multivariate analysis of variance using distance matrices (PERMANOVA). At the end of phase 1, we found no significant difference in the fecal microbiomes of dogs due to breed, sex, body condition, or cohort (pseudo F statistic < 2.7, *P* > 0.05 in all cases) (see Fig. S1A to D in the supplemental material). Likewise, when weighted UniFrac distances of phase 1 samples from dogs of each body condition were tested, we observed no significant difference in the overall microbiome compositions between dogs that were prospectively assigned to either treatment diet (pseudo F < 0.5, *P* > 0.7 in both cases).

10.1128/mBio.01703-16.1 FIG S1  Beta diversity analysis of breed, sex, body condition, and cohort of phase 1 samples using principal-coordinate analysis (PCoA). Download FIG S1, TIF file, 2.1 MB. Copyright © 2017 Li et al. 2017 Li et al. This content is distributed under the terms of the Creative Commons Attribution 4.0 International license.

**FIG 2:**
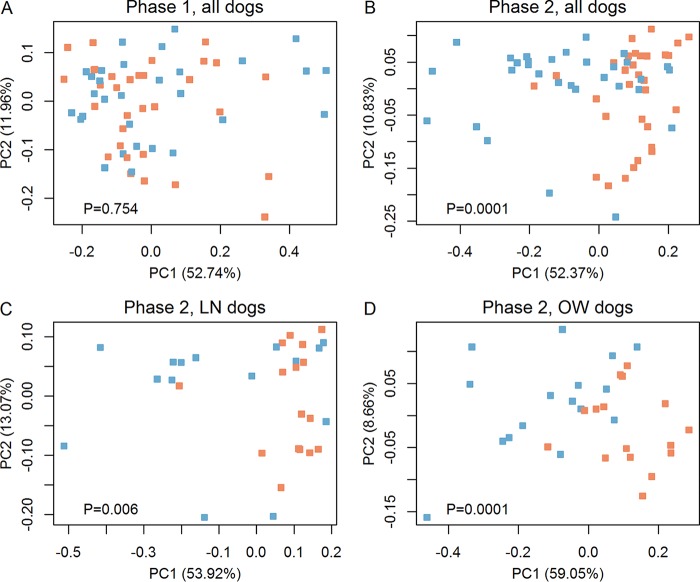
16S rRNA gene surveys revealed dietary effects on gut microbial community. Bacterial beta diversity analysis was performed using principal-coordinate analysis (PCoA) of weighted UniFrac matrices. The percentage of variation explained by the principal coordinates (PC1 and PC2) is indicated on the axes. (A) No clustering was found between dogs prospectively assigned the HPLC versus the LPHC diet at the end of phase 1 (*P* = 0.754). (B) Visible clustering formed between dogs fed the HPLC and LPHC diets at the end of phase 2 (*P* = 0.0001). Dietary influence was stronger in OW dogs (*P* = 0.0001) (D) than in LN dogs (*P* = 0.006) (C). Blue squares represent dogs assigned to the HPLC diet, while orange squares represent dogs assigned to the LPHC diet.

The treatment diets had a significant impact on the fecal communities of all dogs (pseudo F = 13, *P* = 0.0001) (Fig. 2B), which was also obvious in both the LN (pseudo F = 5.0, *P* = 0.006) (Fig. 2C) and the OW (pseudo F = 13, *P* = 0.0001) (Fig. 2D) dogs after phase 2. No cluster was found among base dogs (*P* = 0.754) (Fig. 1A), suggesting that the separation between values from HPLC and LPHC diet-fed dogs was due to their diet. The treatment diets also induced a significant difference in the communities of male and female dogs, regardless of breed or diet (pseudo F = 3.1, *P* = 0.032), though this was driven by differences in the values from OW cohorts (pseudo F = 3.4, *P* = 0.032), as there was no difference in the values from LN cohorts due to sex. Differences between values for Labrador retrievers and Beagles with either body condition were not observed in phase 2. Cohen’s d effect sizes for the diet (HPLC versus LPHC) were computed for OW and LN dogs, with Cohen’s d equal to 0.52 and 0.45, respectively.

Multivariate analysis by linear models (MaAsLin) was performed to find any association of microbial abundance or function with potential confounding factors, such as body weight, food intake, sex, age, cohort, or breed. No significant association was found between the clinical metadata and bacterial abundance and function.

### Differential dietary effects on gut bacterial phyla and families.

We further examined individual bacteria at both the phylum (Fig. 1C) and the family (Fig. 1D) level. Three bacterial phyla, *Firmicutes*, *Bacteroidetes*, and *Fusobacteria*, accounted for 39.32%, 35.22%, and 19.33% of all gut microbes, respectively (Table S1). The HPLC diet-fed dogs had a marked increase in *Firmicutes* but a decrease in *Bacteroidetes* abundances compared with base diet-fed dogs. In comparison, little change was observed in *Firmicutes* or *Bacteroidetes* between LPHC and base diet-fed dogs. The changes in *Fusobacteria* were relatively small among the three dietary groups.

10.1128/mBio.01703-16.2 TABLE S1  Phylum-level abundances of gut bacteria in base, HPLC, and LPHC diet-fed dogs within the OW and LN body condition groups. Download TABLE S1, XLSX file, 0.01 MB. Copyright © 2017 Li et al. 2017 Li et al. This content is distributed under the terms of the Creative Commons Attribution 4.0 International license.

The most abundant families in each of the top three phyla were also examined for their abundance shifts (Fig. 1D; Table S2). In HPLC diet-fed dogs, the abundances of three *Firmicutes* families, *Clostridiaceae*, *Lachnospiraceae*, and *Ruminococcaceae*, increased while those of *Erysipelotrichaceae*, *Veillonellaceae*, and *Lactobacillaceae* decreased compared with abundances in LPHC diet-fed dogs. Remarkably, differential dietary effects on LN versus OW dogs were noticed in the top three *Bacteroidetes* families: *Prevotellaceae* was 4.6 times more abundant in LPHC diet-fed dogs than in HPLC diet-fed dogs in the OW group, but little change was observed in the LN group. The differences in *Paraprevotellaceae* abundances between LPHC and HPLC diet-fed dogs were 1.73-fold versus 2.26-fold in the LN and OW groups, respectively. A decreased abundance of *Bacteroidaceae* was found in LPHC versus HPLC diet-fed dogs in the OW group, but little difference was observed in the LN group.

10.1128/mBio.01703-16.3 TABLE S2  Family-level abundances of gut bacteria in base, HPLC, and LPHC diet-fed dogs within the OW and LN body condition groups. Download TABLE S2, XLSX file, 0.01 MB. Copyright © 2017 Li et al. 2017 Li et al. This content is distributed under the terms of the Creative Commons Attribution 4.0 International license.

### Differentially abundant gut bacterial genera and species.

**FIG 3:**
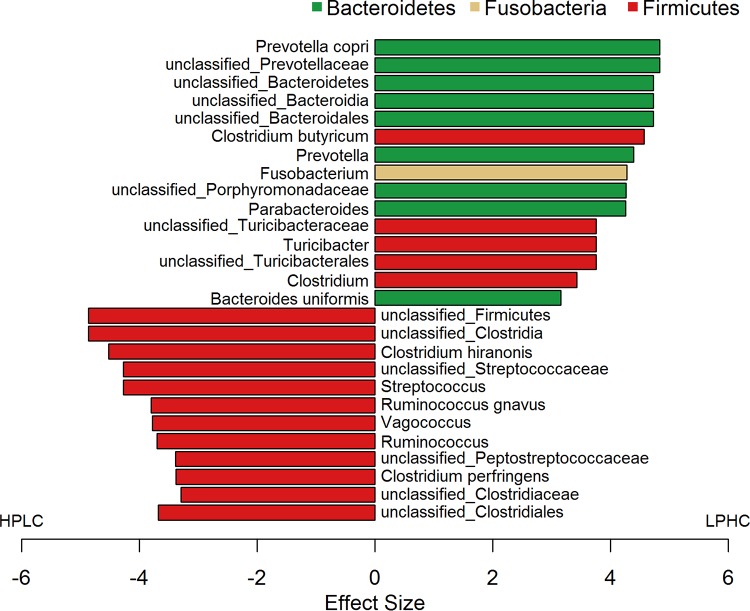
LEfSe was used to compare the abundances of all detected bacterial taxa between HPLC and LPHC diet-fed dogs. The differentially abundant taxa shown in the histogram are significantly different by the Kruskal-Wallis test (FDR < 0.01) and have an LDA score of greater than 3.0. The bacterial taxa associated with positive LDA scores (right) were overrepresented in LPHC diet-fed dogs, while those with negative scores (left) were overrepresented in HPLC diet-fed dogs. All bacteria in the phyla *Bacteroidetes* (green) and *Fusobacteria* (beige) were enriched in LPHC diet-fed dogs, while most bacterial lineages of *Firmicutes* (red) were enriched in HPLC diet-fed dogs.

Linear discriminant analysis (LDA) effect size (LEfSe) analysis was performed to compare the abundances of all detected bacterial taxa between HPLC and LPHC diet-fed dogs, generating both an effect size and a *P* value for each comparison. A total of 27 bacterial lineages were identified as significant by both the Kruskal-Wallis test adjusted for multiple testing (false-discovery rate [FDR], <0.01) and the effect size analysis (LDA score, >3). All *Bacteroidetes* and *Fusobacteria* were overrepresented in LPHC diet-fed dogs, while a majority of *Firmicutes* (>70%) were overrepresented in the HPLC diet-fed dogs (Fig. 3).

**FIG 4:**
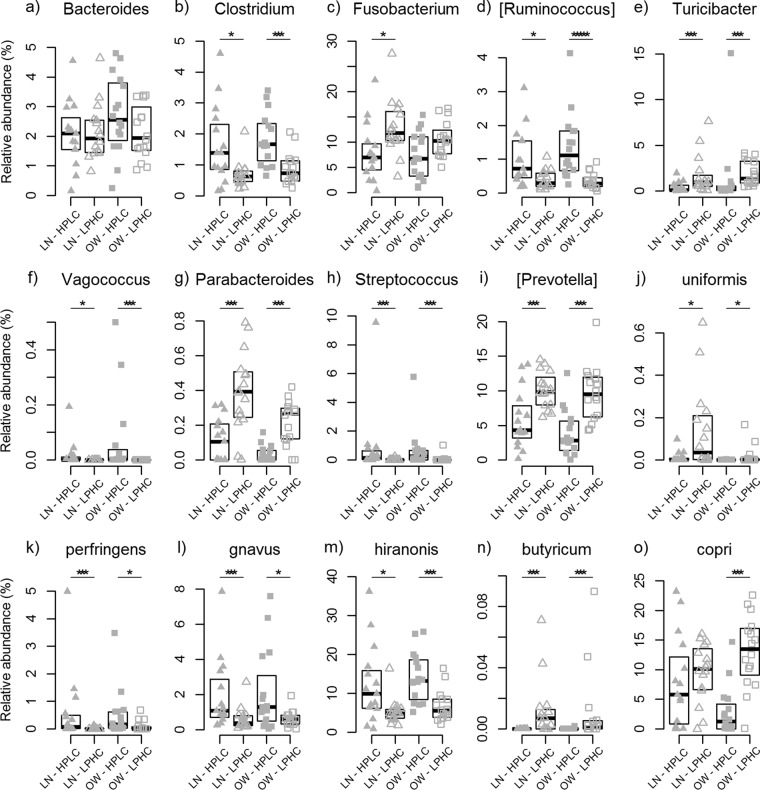
Abundances of selected bacterial genera (a to i) and species (j to o) between HPLC and LPHC diet-fed dogs within each body condition group, LN versus OW, at the end of phase 2. Black lines in the boxplots represent the medians of relative abundance, and the tops and bottoms of the boxes are the first and third quantiles of relative abundances, respectively. The Mann-Whitney *U* test was performed to compare the abundance differences between dietary groups. Levels of statistical significance are denoted as follows: *, *P* < 0.05; **, *P* < 0.01; ***, *P* < 0.001; and ****, *P* < 0.0001.

To investigate which bacteria responded to the dietary influences in LN versus OW dogs, selected bacterial genera and species were examined for their relative abundances by diet and body condition (Fig. 4; Tables S3 and S4). Three species, *Clostridium perfringens*, *Clostridium hiranonis*, and *Ruminococcus gnavus*, were more abundant in HPLC diet-fed dogs than in LPHC diet-fed dogs (Fig. 4k to m), while *Bacteroides uniformis* and *Clostridium butyricum* were overrepresented in LPHC diet-fed dogs (Fig. 4j and n). An increased abundance of *Prevotella copri* in LPHC versus HPLC diet-fed dogs was found only in the OW group, not in the LN group (Fig. 4o). At the genus level, *Clostridium*, *Ruminococcus*, *Vagococcus*, and *Streptococcus* were overrepresented, while *Fusobacterium*, *Turicibacter*, *Parabacteroides*, and *Prevotella* were underrepresented in the HPLC diet-fed dogs, compared with the LPHC diet-fed dogs (Fig. 4b to i). However, *Bacteroides* abundances were not different between the two dietary groups (Fig. 4a).

10.1128/mBio.01703-16.4 TABLE S3  Genus-level abundances of gut bacteria in base, HPLC, and LPHC diet-fed dogs within the OW and LN body condition groups. Download TABLE S3, XLSX file, 0.01 MB. Copyright © 2017 Li et al. 2017 Li et al. This content is distributed under the terms of the Creative Commons Attribution 4.0 International license.

10.1128/mBio.01703-16.5 TABLE S4  Species-level abundances of gut bacteria in base, HPLC, and LPHC diet-fed dogs within the OW and LN body condition groups. Download TABLE S4, XLSX file, 0.01 MB. Copyright © 2017 Li et al. 2017 Li et al. This content is distributed under the terms of the Creative Commons Attribution 4.0 International license.

### B/F and B/P ratios.

**FIG 5:**
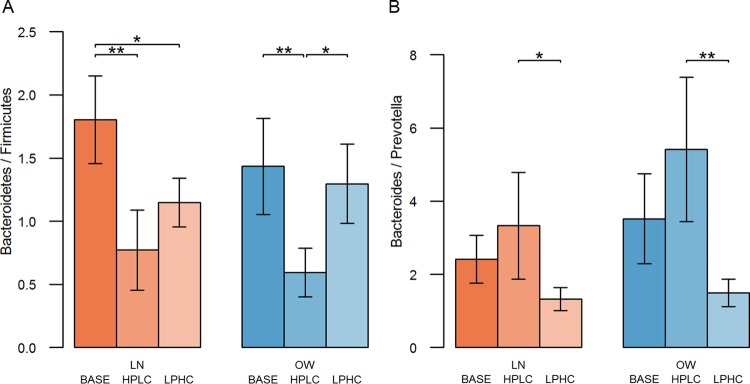
Bar plots of abundance ratios of *Bacteroidetes* to *Firmicutes* (A) and *Bacteroides* to *Prevotella* (B) in each of the three dietary groups, namely, dogs within LN and OW body condition groups fed the base, HPLC, and LPHC diets. Levels of statistical significance are denoted as follows: *, *P* < 0.05, and **, *P* < 0.01.

The *Bacteroidetes*-to-*Firmicutes* ratios (B/F ratios) were not different between base diet-fed LN dogs and base diet-fed OW dogs (*P* > 0.05). Higher B/F ratios were observed in dogs fed carbohydrate-rich diets (i.e., base and LPHC diets) than in dogs fed the protein-rich HPLC diet in the OW group (Fig. 5A, right panel) (*P* < 0.05). However, the difference in B/F ratios between LPHC and HPLC diet-fed dogs diminished in the LN group (Fig. 5A, left panel). In addition, we compared *Bacteroides*-to-*Prevotella* ratios (B/P ratios) for LN and OW dogs. The B/P ratio was significantly higher in HPLC diet-fed dogs than in LPHC diet-fed dogs in both the LN and the OW group (Fig. 5B) (*P* < 0.05), but no difference was found between base diet-fed dogs and dogs of other dietary groups.

### Functional changes in gut microbiota.

**FIG 6:**
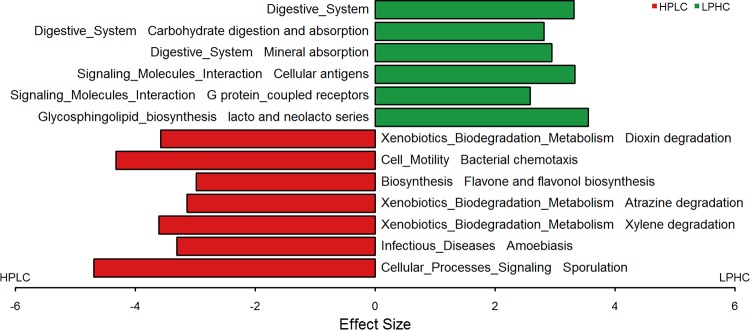
LEfSe was used to compare the abundances of the PICRUSt-predicted KEGG ortholog (KO) functions between HPLC and LPHC diet-fed dogs. The significant KO pathways were selected based on both the Kruskal-Wallis test (FDR < 0.0001) and an LDA score of greater than 2.5. The KO pathways associated with positive LDA scores (green) were overrepresented in LPHC diet-fed dogs, while those associated with negative scores (red) were more abundant in HPLC diet-fed dogs. Pathways in nutrient digestion/absorption and molecular signaling were among those overrepresented in LPHC diet-fed dogs. Pathways involved in xenobiotic biodegradation and flavone biosynthesis were overrepresented in HPLC diet-fed dogs.

To investigate the dietary effect on gut microbial function, phylogenetic investigation of communities by reconstruction of unobserved states (PICRUSt) was performed on the 16S rRNA gene gut bacterial composition data to predict Kyoto Encyclopedia of Genes and Genomes (KEGG) orthologs (KOs) and pathways (26). All predicted KO pathways were subject to LEfSe analysis, and 13 pathways were identified by using selection criteria of an FDR of <0.01 and an LDA score of >2.5 (Fig. 6). Of those pathways, the digestive-system, signaling molecule and interaction, and glycosphingolipid biosynthesis pathways were overrepresented in LPHC diet-fed dogs, while the xenobiotic-biodegradation and metabolism, cell motility, biosynthesis, and cellular processes and signaling pathways were overrepresented in the HPLC diet-fed dogs. In the third level of the KO hierarchy, three degradation pathways (xylene degradation, atrazine degradation, and dioxin degradation), two signal transduction networks (chemotaxis and sporulation), and the flavone and flavonol biosynthesis pathways were overrepresented in HPLC diet-fed dogs, while the carbohydrate digestion and absorption, mineral absorption, and G protein-coupled receptor pathways were overrepresented in LPHC diet-fed dogs. Differentially abundant KOs between the two treatment diets are listed in Table S5.

10.1128/mBio.01703-16.6 TABLE S5  Differentially abundant KEGG orthologs (KOs) found in dogs fed HPLC and LPHC diets. Download TABLE S5, XLSX file, 0.03 MB. Copyright © 2017 Li et al. 2017 Li et al. This content is distributed under the terms of the Creative Commons Attribution 4.0 International license.

## DISCUSSION

There has been a surge of information regarding gut microbiota and its impacts on health over the last decade. HPLC diets have been promoted as an effective body weight management strategy for many years, and potential benefits were reported in both humans and animals (20–23). However, little is known on how the dietary protein-to-carbohydrate ratio influences gut microbiotas in dogs. The five predominant bacterial phyla reported in this study, *Fusobacteria*, *Firmicutes*, *Bacteroidetes*, *Actinobacteria*, and *Proteobacteria*, were similar to those in previous reports (27–29), but relative abundances of each phyla can vary from study to study and likely arise from factors such as breed, sex, age, diet, or even different sampling or sequencing methodologies (27). In this study, we observed that evenness but not richness increased in response to diet manipulation; that is, the abundances of taxa present became more equal rather than community members that may have been undetected in phase 1 expanding in growth. The diversity difference between the two treatment diets was marginally significant in OW dogs (*P* = 0.0562) but not in LN dogs (*P* = 0.7402), suggesting greater dietary effects on OW dogs than on LN dogs. Kittens fed an HPLC diet showed a greater diversity and stability in their gut microbiomes than those fed a diet lower in protein but higher in carbohydrates (30). Our data showed an increased evenness in microbiota in OW dogs fed an LPHC diet compared to that of OW dogs fed an HPLC diet. Cats have a higher protein requirement than dogs. Therefore, the responses to dietary protein and carbohydrate might be different between dogs and cats. It is difficult to fully assess our observations, as the number of cross-sectional studies on the canine microbiome is few in comparison to the number available for humans and other mammals. For instance, community richness in humans has been associated with improvements in metabolic health after bariatric surgery (31) and has been seen to be greater in people consuming traditional diets than in those consuming Westernized diets (16). Thus, it is possible that increased diversity is important or a marker of healthy microbiomes in canines. In either case, the diets used in the present work have an obvious impact on diversity, though it is unknown whether our observations are broadly applicable to other canines or diet compositions without having a greater number of studies to which our data can be compared.

The effect of diet on mammalian gut microbiotas is becoming better understood, as a number of studies have shown shifts in beta diversity in response to changes in diet composition (13). In this study, we demonstrated that HPLC and LPHC diets had significant effects on fecal microbial communities in both LN and OW dogs belonging to two different breeds. Our results appear to be in agreement with those of Handl et al., who observed no obvious clustering of fecal microbiomes between obese and lean groups of pet dogs or research dogs fed a commercial diet for 6 months (32). Our data indicate that even with some degree of overlap in beta diversities visualized by principal-component analysis (PCoA), statistical testing of weighted UniFrac distances demonstrated the significant impact of HPLC and LPHC diets on the compositions of fecal microbiomes not apparent after phase 1. Further, we noted that data for males and females in the OW cohort at the end of phase 2 were significantly different, indicating that the base diet may have masked differences between the sexes during phase 1. Taken together, these results suggest that the dog fecal microbiome may be driven by macronutrient composition, as has been observed in other mammals subjected to changes in diet regimen.

Among the *Firmicutes*, the families of *Clostridiaceae*, *Lachnospiraceae*, and *Ruminococcaceae* were dominant in HPLC diet-fed dogs. The *Clostridiaceae* family included three differentially abundant species, *C. perfringens*, *C. hiranonis*, and *C. butyricum*, while the *Lachnospiraceae* family included *R. gnavus*. The abundances of the three Gram-positive anaerobes, *C. hiranonis*, *C. perfringens*, and *R. gnavus*, in HPLC diet-fed dogs were more than double those in LPHC dogs. Functionally, *C. hiranonis* is thought to be involved in bile acid metabolism (33). Interestingly, in a study with over 150 dogs, *C. perfringens* was found to be present in a high abundance in healthy dogs and is considered a common commensal in dogs (34). *R. gnavus* is one of the “core” bacterial species in human guts (35), where some of its strains are thought to have potential beneficial roles (36, 37) and favor the growth of *Bacteroides* (38). On the other hand, *C. butyricum* was more abundant in LPHC diet-fed dogs than in dogs fed the HPLC diet and is known for its ability to produce a large amount of SCFAs, with many associated health benefits (39). The specific strains of these various organisms were not identified within this study.

Among the *Bacteroidetes*, *Prevotellaceae* and *Paraprevotellaceae* were significantly less abundant in HPLC diet-fed dogs than in LPHC diet-fed dogs, while *Bacteroidaceae* were more abundant in HPLC diet-fed than in LPHC diet-fed OW dogs. At the species level, *P. copri* was significantly underrepresented in HPLC diet-fed OW dogs compared with its levels in base or LPHC diet-fed OW dogs, but little difference was observed in LN dogs. Although a recent study reported a significant association between *P. copri*, rheumatoid arthritis, and the loss of beneficial bacteria in the gut (40), an increase in abundance in *P. copri* was shown to improve glucose metabolism and promote glycogen storage in respond to dietary fiber consumption and the ability to utilize complex polysaccharides (41). This is consistent with our observation of an increased representation of this bacterium in dogs fed carbohydrate-rich diets. Another differentially abundant *Bacteroides* species, *B. uniformis*, was overrepresented in LPHC diet-fed dogs versus HPLC diet-fed dogs. Preclinical evaluations of *B. uniformis* have suggested potential benefits against inflammatory and metabolic disorders (42). In a study of high-fat-diet-induced obesity, administration of *B. uniformis* CECT7771 reduced body weight gain, liver steatosis, liver and serum cholesterol, and triglyceride concentrations and ameliorated metabolic and immune disorders associated with intestinal dysbiosis in obese mice (43). Although *P. copri* has been linked to both beneficial and adverse effects, *B. uniformis* CECT7771 is considered a potential probiotic strain. Further investigation is needed to understand the molecular mechanisms of action of these gut microbes in relation to increased carbohydrate consumptions.

The B/F ratios were not different between base diet-fed OW dogs and base diet-fed LN dogs. Although some studies reported that more *Bacteroidetes* than *Firmicutes* in the gut was correlated with increased metabolic benefits (7, 8, 11), others showed no correlation or even an opposite correlation (44–46). Our observation of a lower B/F ratio in the HPLC diet-fed dogs than in other dogs raised a possibility that a change of B/F ratio in favor of *Firmicutes* might promote weight loss in dogs. At the genus level, we observed an increased B/P ratio in HPLC diet-fed dogs compared with that in LPHC diet-fed dogs, consistent with the previous observations that *Bacteroides* was found to be highly associated with animal protein while *Prevotella* was associated with carbohydrates (16, 18, 41), suggesting a similar link between dietary patterns and gut microbiota compositions in dogs.

Complex interplays between diet, gut microbiota, and signaling cascades may encompass multiple beneficial or harmful effects on the host metabolism and immune health. The HPLC diet-fed dogs showed a greater prevalence of the microbial genes involved in xenobiotic biodegradation and metabolism pathways. A microbial network of genes for weight maintenance was found to be involved in xenobiotic metabolism and degradation, among other things, in humans (11, 47, 48), suggesting a potential association between microbial xenobiotic metabolism and body weight control. In a recent 24-year study of over 124,000 middle-aged or older people, increased intake of food rich in flavonoid were associated with weight maintenance (49). Thus, an overrepresentation of flavone and the flavonol biosynthesis pathway in the gut microbiome may in part explain weight management benefits of the HPLC diets. In LPHC diet-fed dogs, two predicted KO pathways, carbohydrate digestion/absorption and mineral absorption, were overrepresented. This may reflect the enrichment of intestinal bacteria capable of fermenting and utilizing dietary carbohydrates, which in turn increases mineral bioavailability and promotes colonic absorption (50). SCFAs, including butyrate, acetate, and propionate, are produced in abundance during fermentation of dietary fibers by saccharolytic gut microbes, such as *C. butyricum*, and are ligands for G protein-coupled receptors. GRP43, a member of the G protein-coupled receptor family, was shown to be activated by SCFAs to confer beneficial effects on obesity and inflammation in mice (51, 52). Because commonalities between human and dog microbiota are not well defined, further research is needed to better understand the roles of gut microbe-mediated host metabolic signaling in weight management in dogs.

Although the Mann-Whitney U test has often been used in microbiome studies (32, 66), the compositional structure in microbial data may violate some of its assumptions. Novel methods accounting for compositional constraints have been introduced in recent years and should be explored in future studies (67). It is also worth noting that dogs in our study were fed to maintain their body weight, where food was adjusted individually based upon the animal’s weekly body weight. As a consequence, changes in body weight and its possible associations with dietary effects were not observed. It is possible that body weight change may become a confounding factor in pet dogs to whom a fixed or even an extra amount of food is administered by their owners.

In conclusion, our study demonstrated a strong influence of the dietary protein-to-carbohydrate ratio on intestinal microbial compositions and predicted functions and showed that dietary impact was more in evidence in OW dogs than in LN dogs. Consumption of diets high in protein or carbohydrate significantly increased gut microbiota evenness, but not richness, compared to that after consumption of the base diet. Although both treatment diets increased evenness, the changes were due to different microbial profiles. Macronutrient distribution affected the gut bacterial taxonomic profile, mainly within the phyla *Firmicutes* and *Bacteroidetes*. The carbohydrate-rich diets appeared to favor the growth of bacteria such as *B. uniformis* and *C. butyricum* and enrich the pathways in digestion and nutrient absorption. Consumption of a protein-rich diet appeared to increase abundances of *C. hiranonis*, *C. perfringens*, and *R. gnavus*, decrease B/F ratios, and enrich pathways associated with body condition. Our results further suggested that the effects are not breed dependent and are likely to be applicable to a more general population of dogs. More research is needed to understand the complex relationship between diet, gut microbes, and host metabolism.

## MATERIALS AND METHODS

### Animals and study design.

The animal study protocol was approved by the Animal Care and Use Committee of the Nestlé Purina PetCare Company. Thirty-two Labrador retrievers and 32 Beagles, half with a lean or normal (LN) body condition and half with an overweight or obese (OW) body condition, were selected for a two-phase feeding study (Table 1). Dogs with body fat at <34% for Labrador retrievers or 30% for Beagles were considered LN, while those with body fat at >34% for Labrador retrievers or 30% for Beagles were considered OW, as determined by dual-energy X-ray absorption (DEXA; GE Lunar DPXα with EnCore 2011 software). There were 18 males and 14 females in the HPLC diet group, while there were 16 males and 16 females in the LPHC diet group. The mean ages for HPLC and LPHC diet dogs were 5.57 and 5.85 years, respectively. During phase 1, all dogs were fed a commercially available diet (base diet [Purina ProPlan Sport Active 26/16 chicken and rice]) for 4 weeks. At the end of phase 1, dogs in each breed and body condition group were randomized by body fat, age, and sex into two dietary treatment groups: the HPLC and LPHC diet groups. During phase 2, dogs were fed their assigned diets for 4 weeks. Dogs were individually fed to maintain their body weights. The maintenance energy requirement (MER) was estimated using the equation MER = 139 × BW^0.67^ (kilocalories), where BW is the body weight of the dog in kilograms. Dogs were weighed weekly, and the amount of food offered to them was increased or decreased by 5% if their body weight decreased or increased more than 5% over their initial body weight, respectively. Fecal samples were collected, and body fat percentage was assessed using DEXA at the ends of phase 1 and phase 2. All dogs were housed in pairs under similar conditions that included access to toys and other environmental enrichment, which allowed social interaction and exercise. Pairs of dogs within the same treatment group were housed in indoor runs with outdoor access. Dogs were separated during once-daily feeding to allow measurement of individual food intake and during fecal sample collection periods. Dogs had access to water at all times. After the study, the amount of food for the OW dogs was adjusted to target their ideal body condition.

### Diets.

All diets, including one base diet and two treatment diets (HPLC and LPHC) were formulated and manufactured by Nestlé Purina PetCare Company (Table 2). The formulations were created to meet or exceed the maintenance nutrient requirement based upon the guidelines of the Association of American Feed Control Officials. All diets contained animal protein as a primary protein source. The protein level in the HPLC diet was adjusted by replacing grains with plant protein.

**TABLE 2:** Nutritional compositions of the base, HPLC, and LPHC diets

Diet	Content (%)								Calculated ME (kcal/g)^a^
	Moisture	Protein	Carbohydrate	Fat	Dietary fiber			Ash	
					Total	Soluble	Insoluble		
Base	8.09	28.06	33.18	15.8	7.83	0.54	7.29	7.04	3.5
HPLC	7.35	49.38	10.92	14.0	12.49	1.19	11.3	5.85	3.3
LPHC	7.52	25.54	38.80	14.5	8.38	0.58	7.80	5.26	3.5

a ME, metabolizable energy.

### Sample collection and fecal DNA extraction.

Fresh fecal samples were collected within 15 min of defecation at the end of each phase and were immediately frozen and stored at −80°C until use. Fecal bacterial DNA was extracted using the PowerFecal DNA isolation kit (Mo Bio Laboratories, Inc., Carlsbad, CA) by following the manufacturer’s protocol. Fecal DNA was quantified using the PicoGreen assay (Thermo Fisher Scientific, Waltham, MA).

### **16S rRNA gene V3**-**V4 region sequencing.**

16S rRNA gene library preparation was performed according to Illumina’s 16S metagenomic sequencing library preparation guide. The sequences for the 16S rRNA amplicon PCR forward and reverse primers were 5′TCGTCGGCAGCGTCAGATGTGTATAAGAGACAGCCTACGGGNGGCWGCAG and 5′GTCTCGTGGGCTCGGAGATGTGTATAAGAGACAGGACTACHVGGGTATCTAATCC, respectively (53). Illumina sequencing was performed using an Illumina MiSeq sequencer at MOgene, LC (St. Louis, MO, USA). A MiSeq reagent kit (v3) was used with the 600-cycle format to generate 300-bp paired-end (PE300) sequences. 

### Sequence processing.

An average of 167,500 PE300 sequences was generated for each sample. Data processing and cleansing were performed using mothur (version 1.34.3) (54). Briefly, paired-end reads were aligned and joined into contigs and filtered to remove sequences with ambiguous bases, contigs shorter than 200 bp or longer than 500 bp, homopolymers of eight or more or those that failed to align with the appropriate 16S rRNA variable region of the Silva bacterial reference data set (release 123) (55). Sequences were then demultiplexed by sample groups using barcode sequences. Samples with less than 8,000 sequences were excluded from further analysis.

### Bioinformatic analysis.

Chimeric sequences were identified using the UCHIME (version 4.2) algorithm with both *de novo* and reference-based detection methods (56) and were subsequently removed. Sequences were grouped into operational taxonomic unites (OTUs) using the UCLUST-based closed-reference OTU picking method implemented in QIIME (version 1.9.1) (57, 58). Sequences that shared 97% identity were assigned to the same OTU group. Taxonomy was assigned using the predefined taxonomy map derived from the Greengenes database (August 2013 release) (59). Putative bacterial metagenomic functions were imputed using phylogenetic investigation of communities by reconstruction of unobserved states (PICRUSt) on the 16S rRNA gene abundance data (26). Although PICRUSt has not been validated in dogs, the mean score for the nearest sequenced taxon index (mean NSTI = 0.087 ± 0.009 SD), which measures the dissimilarity between reference genomes and the predicted canine metagenomes, is comparable to that from human samples. The OTU table was normalized by dividing each OTU by the known or predicted 16S rRNA copy number abundances before metagenomic function prediction.

Bacterial compositional data in the OTU table were normalized by calculating relative abundance, where each feature count was divided by the total sequence count in each sample. An additional low-abundance filter was applied to remove features whose relative abundances did not exceed 0.01% in any sample.

Both alpha and beta diversity indexes were calculated using QIIME (58). The OTU table was first rarefied by subsampling the full OTU table to a depth coverage of 7,000 sequences per sample for 100 iterations. Two alpha diversity indexes, the Faith's phylogenetic diversity index and Simpson’s evenness, were calculated for each subsampled OTU table, and the sample mean for each metric was taken. Weighted UniFrac (60) distance matrices and principal coordinates for each sample were computed.

### Statistical analysis.

Differentially abundant taxa and Kyoto Encyclopedia of Genes and Genomes (KEGG) pathways were identified using the linear discriminant analysis (LDA) effect size (LEfSe) software (61). Relative abundances of all features were first compared by using the nonparametric Kruskal-Wallis rank sum test, and each statistically significant feature was further subjected to effect size estimation using LDA. False-discovery rates (FDRs) were calculated to adjust for multiple testing errors. Associations between clinical metadata and bacterial abundance data were tested using multivariate analysis by linear models (MaAsLin) (T. Tickle, L. Waldron, Y. Lu, and C. Huttenhower, unpublished data).

Permutational multivariate analysis of variance (PERMANOVA, PRIMER v6) (62) was performed to test whether two groups (e.g., HPLC and LPHC diet-fed dogs) were significantly different on a beta diversity distance matrix using 10,000 permutations. The Mann-Whitney *U* test was performed to compare two groups of samples for alpha diversities and bacterial abundances. The effect size for the diet fed to OW dogs or LN dogs was measured by calculating the absolute value of Cohen’s d effect size on individual taxa (63, 64). The means were then taken as the dietary effect size for OW or LN dogs. The differences in the B/F and B/P abundance ratios among the three dietary groups were tested using ANOVA, followed by Tukey’s *post hoc* tests to identify groups that were significantly different from each other. All statistical computing was performed in R (R Core Team, version 3.2.1). Some graphs were generated using the R scripts adapted from the work of Scher et al. (40).

### Accession number(s). 

Sequencing data for the 16S rRNA sequences have been deposited in the NCBI Sequence Read Archive (SRA) under accession number SRP095473.
