# Supplementary figures and images for: Effects of the Dietary Protein and Carbohydrate Ratio on Gut Microbiomes in Dogs of Different Body Conditions
Source: mBio. 2017 Jan 24;8(1):e01703-16. doi: 10.1128/mBio.01703-16 (PMC5263242; doi:10.1128/mBio.01703-16)

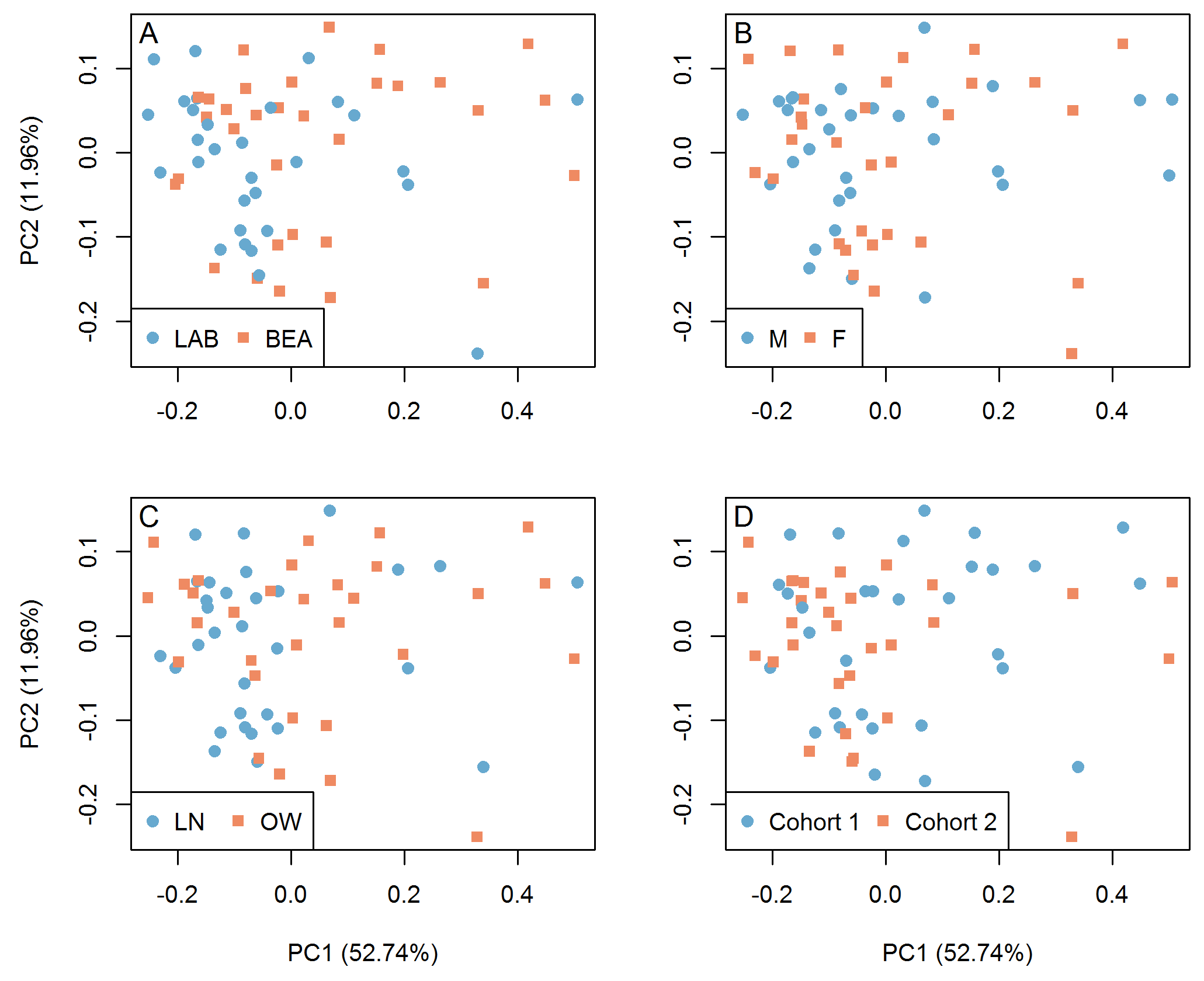

Supplement: FIG S1 [file mbo001173165sf1.tif]
